# What influences psychological functioning in patients with mood disorders? The role of clinical, sociodemographic, and temperamental characteristics in a naturalistic study

**DOI:** 10.1186/s12991-022-00428-9

**Published:** 2022-12-24

**Authors:** Matteo Di Vincenzo, Gaia Sampogna, Bianca Della Rocca, Carlotta Brandi, Emiliana Mancuso, Lorenzo Landolfi, Antonio Volpicelli, Arcangelo Di Cerbo, Andrea Fiorillo, Mario Luciano

**Affiliations:** Department of Psychiatry, University of Campania “L. Vanvitelli”, Largo Madonna Delle Grazie, 80139 Naples, Italy

**Keywords:** Functioning, psychological, Bipolar disorder, Depression, Quality of life, Affective temperaments, Cognition, Impulsivity, Suicidality

## Abstract

**Background:**

The present study aims to assess clinical and psychological correlates of psychological functioning in patients with mood disorders, in a naturalistic setting. In particular, we aimed to describe which sociodemographic, clinical, and temperamental dispositions are more frequently associated with poor psychological functioning, and to describe the association between cognitive and psychological functioning in euthymic patients with major depression and bipolar disorder.

**Methods:**

Inclusion criteria were as follows: (1) diagnosis of major depression, or bipolar disorder type I or II; (2) age between 18 and 65 years; and (3) being in a stable phase of the disorder. Patients’ psychiatric symptoms, quality of life, affective temperaments, and impulsivity were investigated with validated assessment instruments.

**Results:**

166 patients have been recruited, mainly female (55.4%), whose mean age was 47.1 ± 14.2 years. 42.6% of individuals reported a diagnosis of major depression. According to regression analyses, poor cognitive performance (*p* < 0.05), reduced perceived quality of life (p < .0001), lifetime suicide attempts (*p* < 0.01), and increased trait-related impulsivity (*p* <0 .001) strongly correlated with poor psychological functioning. Moreover, cyclothymic and irritable dispositions were also associated with poor social functioning (*p* < 0.01), whereas hyperthymic affective disposition was associated to a better psychological performance (p < 0.01).

**Conclusions:**

Our results support the evidence that patients with mood disorders should be assessed for psychological functioning and affective dispositions, to identify patients at higher risk to develop worse long-term outcomes and to develop targeted interventions.

## Introduction

Mood disorders (MDs) are among the most frequent psychiatric disorders, with a prevalence of major depressive disorder (MDD) ranging from 13 to 20% in the general population [4, 24] and of bipolar disorder (BD) ranging from 3.1 to 8% [48, 71]. Moreover, mood disorders are associated with a significant personal and social burden, being listed among the top-ten leading causes of disability and of premature death by the World Health Organization [20]. More than half of patients with MDs present high comorbidity rates with other psychiatric and physical disorders, including anxiety, alcohol use disorders, chronic pain, and metabolic and cardiovascular disorders [13, 53, 70]. Both depressive and manic/hypomanic symptoms are associated to functional impairment and reduced quality of life (QoL), with difficulties in many areas [43, 63], including work [21], family, and social functioning [22, 39, 44, 66]. The level of impairment is comparable in individuals with MDD and BD during depressive phases and it is worse than that observed in most chronic physical illnesses [11, 29].

Symptoms have been usually considered the primary target of psychiatric treatments [42, 69]. However, patients’ QoL remains unsatisfactory even after clinical remission [18] in a vast majority of patients, including asymptomatic patients and those with residual or subthreshold symptoms [46].

psychological impairment in patients with MDD and BD has been correlated to a variety of factors, including clinical, sociodemographic, and psychological aspects. In particular, from a clinical viewpoint, psychological functioning is influenced by symptom severity, illness duration, presence of psychotic symptoms during acute phases, use of psychotropic medications [41], and cognitive deficits [57, 58], such as attention, executive functions, learning, and memory [7, 38, 46]. In particular, information processing speed, learning and memory impairments, and executive dysfunctions are compromised in patients with MDD and BD [40, 45, 68].

Sociodemographic characteristics associated with higher levels of functional impairment in patients with MDs include older age, male gender, and belonging to ethnical minorities [3, 5]. The psychological dimensions which could affect psychological functioning of patient s with MDs include coping skills, hopelessness, mental rigidity, and problem-solving strategies [54, 78]. Among psychological domains, only rarely the role of affective temperaments in influencing patients’ psychological functioning has been explored. Temperamental dispositions have been described as stable parts of personality [74], which reflect interpersonal styles, energy level, and sensitivity to stimuli. Affective temperaments, as conceptualized by [2], are the anxious, irritable, cyclothymic, hyperthymic, and depressive one [1]. The only dimension of functioning that has been associated to affective disposition is neurocognitive functioning. Russo et al. [60] reported that the presence of cyclothymic and hyperthymic dispositions is associated to a better cognitive performance, and that depressive and anxious predominant dispositions were associated to poor cognitive skills. Considering the relationship between cognitive and psychological functioning, we can only indirectly assume that some affective dispositions can be associated with a better psychological functioning. However, at the moment, no study has directly explored the association between affective dispositions and psychological functioning of individuals with MDs.

Despite levels of psychological impairment in individuals with MDs varies according to the duration and severity of the illness, deficits in global functioning are not always temporally confined to acute episodes, with persistence of psychological impairment over time [15, 37, 73] Impairment in social functioning may persist for years after the resolution of an affective episode, depending on the thoroughness (i.e., with vs. without residual symptoms) and stability (i.e., persistence over time) of the remission.

Currently, research on psychological functioning in patients with MDs is still limited, and few evidence is available on the nature and the extent of psychological impairments in individuals with MDs; differences in methodologies greatly contributed to the heterogeneity of results. Moreover, the clinical characterization of patients with MDs presenting a significant psychological impairment is still missing. One possible major contribution to the paucity of available data is the fact that a clear definition of psychological functioning is lacking with regard to patients with MDs. Currently, several definitions of psychological functioning exist, with their common elements comprising both psychological and social functioning. In this paper we adopted the definition formulated by Xhang et al. (2016) who described psychological functioning as the ability of an individual with MDs to create effective relationships with others and the society in a mutually pleasing manner, and the ability to achieve a healthy life independently.

The aim of the current study is to assess the clinical and psychological correlates of psychological functioning in patients with mood disorders. In particular, we aimed to describe which sociodemographic, clinical, and temperamental dispositions are more frequently associated with poor psychological functioning and to describe the relationship between cognitive and psychological functioning in patient with MDD and BD.

## Methods

This study was carried out at the Department of Psychiatry of the University of Campania “Luigi Vanvitelli”. Patients were recruited if they (1) had a diagnosis of MDD or BD type I or II; (2) aged between 18 and 65 years; and (3) were in a stable phase of the disorder. Informed consent was obtained by all study participants. Patients were excluded from this study if they presented comorbid neurological diseases or drug and alcohol dependence. This study was approved by the Local Research Ethic Committee (Number: N001567/28.01.2018).

## Procedures

### Psychopathological assessments

Sociodemographic and clinical characteristics were recorded thought an ad hoc schedule.

The Hamilton Depression Rating Scale (HAM-D) [23] was adopted to assess severity of depressive symptoms. The HAM-D includes 17 items. Of these, 8 items are scored from 0 (absent) to 4 (severe), while nine are scored from 0 to 2. The total score is performed by the sum of the items’ scores and ranges from 0 to 52 points.

Manic symptoms were assessed with the Young Mania Rating Scale (YMRS) [77]. YMRS includes eleven items, assessing symptoms mood, mobility, sexual desire, sleep, irritability, speech, flight of ideas, grandiosity, aggressive behaviors, appearance, and insight. Seven items are rated on a 5-point Likert scale (from 0 to 4), while four items were rated on a 9-point Likert scale (from 0 to 8).

QoL was assessed thought the Manchester Short Assessment of Quality of Life (MANSA) [56], a 12-item instruments which assess satisfaction across different life domains. Items are assessed on a 7-point Likert scale (1–7).

The brief version of the Munster Temperament Evaluation of the Memphis, Pisa, Paris and San Diego (b-TEMPS-M) was administered to assess affective dispositions. The b-TEMPS-M is a 35-item questionnaire. Each item is scored from 1 to 5 (1 = “not at all”; 2 = “a little”; 3 = “moderately”; 4 = “much”; 5 “very much”) [16]. Five subscales can be calculated, corresponding to the five affective temperaments. Cronbach’s alpha coefficients for subscales were all above 0.8, Kaiser-Meyer-Olkin (KMO) was 0.914.

Trait-related impulsiveness was assessed through The Barratt Impulsiveness Scale (BIS-11) [17]. BIS-11 items are scored on a 4-point Likert scale (1 = rarely, 4 = almost always/always). Higher BIS-11 total scores indicate higher impulsivity traits.

Cognitive functioning was assessed through the brief version of the Measurement and Treatment Research to Improve Cognition in Schizophrenia (MATRICS) Consensus Cognitive Battery (MCCB), which included the Trail Making Test–part A (TMT-A), the Brief Assessment of Cognition in Schizophrenia: Symbol Coding (BACS), and the Category Fluency-Animal Naming [30].

psychological functioning was assessed through the Personal and Social Performance Scale [49] which assesses patients’ functioning across four dimensions (social activities, interpersonal relationships, self-care, aggressive behaviors). Based on ratings on the four dimensions a total score can be attributed to score the overall patient’s functioning, ranging from 0 to 100, with higher scores indicating higher functioning.

### Statistical analyses

Sociodemographic and clinical characteristics, and total scores for assessment instruments were assessed through descriptive statistics. Sample was then divided according to the diagnosis (i.e., MDD vs. BD). T-Student test or *χ*2 was used to test differences among groups. Pearson correlation analyses were adopted in order to assess the association between psychological functioning and continuous clinical variables and total scores. Kendall’s rank analyses were performed to assess correlations between psychological functioning and dichotomous variables. Linear regression analyses were performed, using PSP total score as independent variable. Those variables statistically significant at the univariate analyses were included as covariates. The level of statistical significance was set at *p* < 0.05.

## Results

### Sociodemographic and clinical characteristics

A total of 166 patients were included in this study (Table 1). Half of recruited sample (55.4%) was female, with a mean age of 47.1 ± 14.2 years. 57.4% of them reported a diagnosis of bipolar disorder, with a mean duration of illness of 16.2 ± 13.8 years. Psychotic symptoms during affective episodes were reported by 29% of the sample, while 36 patients had at least one suicide attempt lifetime, while 25.6% of the sample reported a seasonal pattern. Patients reported a mean score of 22.4 ± 6.5 for the depressive affective disposition, 18.6 ± 5.2 for the hyperthymic one, 18.9 ± 7.3 for the anxious subscale, 22.8 ± 8.2 for the cyclothymic subscale, and 16.9 ± 7.6 for the irritable one. Mean score at PSP was 70.3 ± 19.1 and 37.5 ± 134 at B-MCCB symbol coding, 19.0 ± 6.8 at B-MCCB animal naming, and 48.3 ± 20.6 at B-MCCB trial making test A.

**Table 1:** Sociodemographic and clinical characteristics of the sample

	Total sample (*N* = 166)
Age (M ± DS)	47.7 (14.1)
Gender, M, % (*N*)	44.6 (74)
Living situation, with partner yes % (*N*)	50.0 (83)
Years of education (M ± DS)	12.9 (3.7)
Employed, yes, % (*N*)	45.2 (75)
Duration of illness (M ± DS)	16.2 (13.8)
Diagnosis, % (*N*)	
Bipolar disorder	57.4 (93)
Major depression	42.6 (69)
Suicide attempts, yes, % (*N*)	21.8 (36)
Seasonality, yes, % (*N*)	25.6 (42)
Presence of psychotic symptoms during acute phases, yes, % (*N*)	29. (42)
Aggressive behaviors, yes, % (*N*)	23.0 (37)
BIS-11, total score	78.3 (15.5)
BIS11, Attentional impulsiveness subscale	19.22 (4.1)
BIS11, Motor impulsiveness subscale	29.72 (9.0)
BIS11, Non-planning impulsiveness subscale	29.4 (4.7)
Depressive affective temperament	22.4 (6.5)
Hyperthymic affective temperament	18.6 (5.2)
Anxious affective temperament	18.9 (7.3)
Cyclothymic affective temperament	22.8 (8.2)
Irritable affective temperament	16.9 (7.6)
HAM-D, total score	8.5 (9.7)
YMRS, total score	3.0 (6.6)
MANSA, total score	3.8 (1.1)
PSP, total score, M (SD)	70.3 (19.1)
B-MCCB, symbol coding, M (SD)	37.5 (13.4)
B-MCCB, animal naming, M (SD)	19.0 (6.8)
B-MCCB trial making test A, M (SD)	48.3 (20.6)

*MANSA*: Manchester Short Assessment of Quality of Life, *B-MCCB* Brief MATRICS Consensus Cognitive Battery, *PSP* Personal and Social Performance Scale, *BIS-11* Barratt Impulsiveness Scale, *HAM-D* Hamilton Depression Rating Scale, *YMRS*: Young Mania Rating Scale

### Univariate analyses

Compared to patients with major depression, those suffering from bipolar disorders showed a longer duration of illness (20.7 ± 13.2 vs. 10.9 ± 12.5, *p* < 0.0001), higher levels of impulsivity (BIS-11 total score 82.9 ± 16.0 vs. 71.6 ± 12.1), lower HAM-D total score (6.3 ± 11.5 vs. 11.3 ± 5.7, *p* < 0.001), and higher YMRS total score (4.6 ± 8.2 vs. 0.7 ± 1.3). Moreover, they presented more frequently a seasonal pattern (39.8% in patients with bipolar disorder vs. 13.2% in patients with major depression, *p* < 0.0001), history of suicide attempts (26.9% vs. 13.2%, *p* < 0.05), and psychotic symptoms during acute phases (43% vs. 3.8%, *p* < 0.0001). Reduced mean score of anxious affective temperament was reported in patients with bipolar disorders, compared to those with major depression (17.1 ± 6.2 vs. 21.4 ± 7.9, *p* < 0.0001) (Table 2). No statistical differences were detected between the two diagnostic groups with respect to psychological functioning (Table 2).

**Table 2:** Sociodemographic and clinical characteristics of the sample, according to diagnosis

	Major depression (*N* = 69)	Bipolar disorder (93)
Age (M ± DS)	48.4 (14.7)	47.7 (13.5)
Gender, M, % (*N*)	33.3 (23)	52.7 (49)
Living situation, with partner yes % (*N*)	28.8 (39)	43.0 (40)
Years of education (M ± DS)	12.2 (3.8)	13.6 (3.5)
Employed, yes, % (*N*)	37.7 (26)	51.6 (48)
Duration of illness (M ± DS)	10.9 (12.5)	20.7 (13.2)^****^
Suicide attempts, yes, % (*N*)	13.2 (9)	26.9 (25)^*^
Seasonality, yes, % (*N*)	6.0 (4)	39.8 (37)^****^
Presence of psychotic symptoms during acute phases, yes, % (*N*)	3.8 (2)	43.0 (40)^****^
Aggressive behaviors, yes, % (*N*)	19.1 (13)	23.6 (21)
BIS-11, total score (M ± DS)	71.6 (12.1)	82.9 (16.0)^****^
BIS11, Attentional impulsiveness subscale (M ± DS)	17.6 (3.4)	20.3 (4.3)^****^
BIS11, Motor impulsiveness subscale (M ± DS)	32.4 (9.3)	25.9 (7.1)^****^
BIS11, Non-planning impulsiveness subscale (M ± DS)	28.1 (5.0)	30.1 (4.2)^****^
Depressive affective temperament (M ± DS)	22.5 (6.5)	22.1 (6.6)
Hyperthymic affective temperament (M ± DS)	18.2 (4.8)	19.9 (5.5)
Anxious affective temperament (M ± DS)	21.4 (7.9)	17.1 (6.2)^****^
Cyclothymic affective temperament (M ± DS)	23.2 (8.6)	22.6 (8.0)
Irritable affective temperament (M ± DS)	15.6 (6.4)	17.6 (8.1)
HAM-D, total score (M ± DS)	11.3 (5.7)	6.3 (11.5)^***^
YMRS, total score (M ± DS)	0.7 (1.3)	4.6 (8.2)^****^
MANSA, total score (M ± DS)	3.7 (1.1)	3.8 (1.0)
PSP Total score (M ± DS)	71.3 (17.2)	69.7 (20.9)
B-MCCB, symbol coding, M (SD)	35.7 (12.7)	41.0 (14.3)
B-MCCB, animal naming, M (SD)	19.4 (7.4)	18.9 (5.4)
B-MCCB trial making test A, M (SD)	49.5 (19.8)	46.1 (22.7)

*MANSA* Manchester Short Assessment of Quality of Life, *B-MCCB* Brief MATRICS Consensus Cognitive Battery, *PSP*: Personal and Social Performance Scale, *BIS-11*: Barratt Impulsiveness Scale, *HAM-D* Hamilton Depression Rating Scale, *YMRS* Young Mania Rating Scale

*p < .05

^**^p < .01

^***^p < .001

^****^p < .0001

### Correlation analyses

At correlation analyses (Table 3), factors inversely associated with PSP total score with the strongest level of significance (*p* < 0.0001) were B-MCCB Trial Making Test A score, BIS-11 total score and all BIS-11 subscales (motor, attentional and non-planning impulsiveness), irritable affective temperament, HAM-D total score, and presence of delusions and/or hallucinations during acute phases. Other factors inversely correlated with PSP total score were cyclothymic affective temperament, suicide attempts (*p* < 0.001), duration of illness, anxious affective temperament, and YMRS total score (*p *< 0.05). Factors positively associated with PSP total score were MANSA total score (*p* < 0.0001), B-MCCB animal naming score (*p* < 0.001), and presence of hyperthymic temperament (*p* < 0.01).

**Table 3:** Correlations analyses

	PSP total score
Duration of illness	− 0.179^*^
B-MCCB, symbol coding, M (SD)	0.163
B-MCCB, animal naming, M (SD)	0.432^***^
B-MCCB trial making test A, M (SD)	− 550^****^
BIS11, Attentional impulsiveness subscale	− 0.543^****^
BIS11, Motor impulsiveness subscale	− 0.592^****^
BIS11, Non-planning impulsiveness subscale	− 0.384^****^
BIS11, Total score	− 0.603^****^
Depressive affective temperament	− 0.365^****^
Hyperthymic affective temperament	0.098^****^
Anxious affective temperament	− 0.189^*^
Cyclothymic affective temperament	− 0.231^***^
Irritable affective temperament	− 0.336^****^
HAM-D, total score	− 0.356^****^
YMRS, total score	− 0.189^*^
MANSA, total score	0.547^****^
Presence of psychotic symptoms during acute phases	− 0.324^****^
Seasonality, yes	− 0.139
Presence of suicidal attempts lifetime	− 0.240^***^
Diagnosis of bipolar disorder	− 0.041
Diagnosis of unipolar disorder	0.41

*MANSA* Manchester Short Assessment of Quality of Life, *B-MCCB*: Brief MATRICS Consensus Cognitive Battery; *PSP* Personal and Social Performance Scale, *BIS-11* Barratt Impulsiveness Scale, *HAM-D* Hamilton Depression Rating Scale, *YMRS* Young Mania Rating Scale

^*^p < .05

^**^p < .01

^***^p < .001

^****^p < .0001

### Multivariate analysis

According to the linear regression model (Table 4), the likelihood to have a lower PSP total score was increased by the following: (1) the presence of suicidal attempts lifetime (B = − 1687; *p* < 0.01); (2) lower B-MCCB, animal naming, score (*B* = 0.680, *p* < 0.05), and B-MCCB trial making test *A* score (*B* = − 179, *p* < 0.05); (3) lower BIS-11 total score (*B* = 0.665, *p* < 0.001); and (4) presence of cyclothymic (*B* = − 0.343, *p* < 0.01) and irritable affective temperaments (B = − 0.819, *p* < 0.01). Moreover, hyperthymic affective temperament (*B* = 1.24, *p* < 0.01) and higher MANSA total score (*B* = 9.15, *p* < 0.00001) are associated to higher PSP total score.

**Table 4:** Multivariable regression models

	OR	95% CI	
		Lower bound	Upper bound
Presence of psychotic symptoms during acute phases	− 0.521	− 14,694	13,653
Presence of suicidal attempts lifetime	− 1687**	− 14,007	10,632
Diagnosis of bipolar disorder	− 15,289	− 27,976	− 2602
Diagnosis of unipolar disorder	− 7.876	− 10,432	− 1824
B-MCCB, animal naming, M (SD)	0.680*	− 0.890	1750
B-MCCB trial making test A, M (SD)	− 0.179*	− 0.297	0.456
Duration of illness	0.007	− 0.426	0.440
BIS11, Total score	0.665***	0.110	1220
Depressive affective temperament	− 0.071	− 1374	1231
Hyperthymic affective temperament	1244**	− 0.019	2469
Anxious affective temperament	− 0.005	− 0.923	0.914
Cyclothymic affective temperament	− 0.343**	− 0.653	1038
Irritable affective temperament	− 0.819**	− 1159	1797
HAM-D, total score	− 0.389	− 1080	0.303
MANSA, total score	9155****	2362	15,947
YMRS, total score	− 1673	− 3462	0.116

*MANSA* Manchester Short Assessment of Quality of Life , *B-MCCB* Brief MATRICS Consensus Cognitive Battery, *PSP* Personal and Social Performance Scale, *BIS-11 *Barratt Impulsiveness Scale; *HAM-D* Hamilton Depression Rating Scale, *YMRS*: Young Mania Rating Scale

^*^p < .05

^**^p < .01

^***^p < .001

^****^p < .0001

## Discussion

This is one of the few studies extensively assessing clinical and psychological correlates of psychological functioning in a sample of patients with affective disorders. Moreover, the possible relationship among the five affective predominant dispositions and psychological functioning in individuals with MDs has been investigated only rarely. We have recruited only stable patients, considering that most of the available evidence, with some exceptions, has been collected in patients presenting affective symptoms to a various degree of severity. It has to be noted that affective temperaments reporting can be influenced by affective symptoms, especially in patients with BDs, during active phases of the disorder.

With regard to the first research aim (i.e., which clinical features are associated to poor psychological functioning?), we found that a poor cognitive performance, a reduced perceived quality of life, presence of suicide attempts lifetime, and increased trait-related impulsivity were strongly correlated with a poor psychological functioning.

The evidence that neurocognitive impairment limits creativity, work performance, QoL, and self-esteem has been reported mainly in individuals with schizophrenia [19, 50, 51]. However, little is known about possible effects of neurocognitive deficits in individuals with MDs [46, 76]. The few studies carried out in patients with active affective symptoms [21] or with residual affective symptoms [61] reported that the association between cognitive and psychological functioning could be biased by the presence of depressive or manic/hypomanic symptoms. The persistence of this association in patients without active affective symptoms, reported by the present study, is rather new and suggests that these deficits, especially in speed of processing, are an enduring component of the neuropsychopathology of affective disorders, and not merely manifestations of acute illness. As such, they could be present even before the onset of the first episode of illness and could predict the onset of an affective disorders [38].

In our study, patients with a higher perceived QoL showed a higher psychological functioning. This finding is consistent with Baune et al. [6] and with Knight et al. [35], confirming that impairment in QoL has detrimental effects not only on patients perceived outcomes but also on the overall functioning, including work, social, and affective functioning [10] also in euthymic patients. It has been reported that psychological functioning and QoL are deeply interconnected, with quality of life influencing overall functioning and vice versa. In fact, a reduced psychological functioning is associated with poor working skills and productivity [5], reduced social contacts, and increased feelings of loneliness, with a significant impact on individuals’ QoL [27, 36, 37]. Conversely, dissatisfaction in several aspects of life (i.e., work, family, social life) could affect occupational competitiveness and patients’ motivation to be engaged in social and leisure activities and to maintain regular contacts with family members and other relevant others, thus affecting overall psychological functioning [8, 28, 35].

Moreover, in our sample higher levels of trait-related impulsivity strongly reduced patients’ psychological functioning. This association, which has only rarely been investigated, could be mediated by the fact that high levels of trait-related impulsiveness are associated to a worse long-term outcome and to an increased illness chronicity, leading to a reduced psychological functioning [59]. In fact, in patients with bipolar disorder, trait-related impulsiveness has been associated to an earlier age at onset, increased risk of suicide attempts and higher number of relapses [50, 67], reduced time in euthymic phase [12], more frequent rapid cycling course [14], and substance behaviors [64]. Moreover, impulsivity negatively affect long-term outcome in patients with MDD also, by increasing suicidality [34, 47, 75] substance misuse and mood instability [25, 26]. In particular, impulsivity has been reported to be a predictive factor for future suicidal attempts in patients with mood disorders, [50].

In our study, we reported a significant association among psychological functioning and affective temperaments. In particular, cyclothymic and irritable dispositions were associated to a reduced psychological functioning, whereas a predominant hyperthymic affective disposition with better psychological performances. Affective temperaments have been also associated with different psychopathological dimensions in patients with affective disorders [16, 41]; cyclothymic temperament is usually associated with clinically relevant and persistent mood fluctuations and levels of energy [52], while the irritable disposition is associated with impulsivity and anger. These two affective dispositions are generally associated to a worse outcome, but only rarely their relationship with psychological functioning has been explored. The presence of a hyperthymic predominant disposition usually implies the presence of high energy levels, positive thinking, ambition, confidence, increased social abilities, and increased creativity. Patients with this affective disposition could present, therefore, reduced illness severity and increased coping skills to deal with environmental stressors [31, 55].

Of note, both at correlation analyses and at multivariate analyses, psychiatric diagnoses were not statistically associated with global functioning. This suggests that during euthymic phases of the disorder, global levels of functioning do not significantly differ among patients with MDD and BD. This result further highlight that mood disorders could belong to a broad affective spectrum, in which affective dispositions and psychopathological and psychological domains delineate multiple complex clinical phenotypes. The complex interplay among these factors should guide clinicians toward a better clinical characterization [43, 57, 65]. Moreover, our results suggest that a trans-diagnostic approach to mental disorders should be preferred to a rigid categorical approach [33, 62].

Our study has several limitations. First, patients were recruited only in one site. Moreover, the sample size is relatively small. These two factors limit the generalizability our findings, which should be replicated in larger sample sizes. Moreover, we did not include a control group of patients suffering from mental disorders different form affective ones. Third, the cross-sectional design of the study did not allow us to investigate cause–effect relationships. Another possible limitation of the present study is that affective temperaments were detected with a self-reported questionnaire. However, the TEMPS is the most adopted assessment instruments and objective measures to evaluate affective dispositions are not available. Moreover, in our study several clinical characteristics have been retrospectively assessed (i.e., age at onset, number of previous relapses, presence of psychotic symptoms during affective phases, and so on). However, we tried to reduce this recall bias by using a structured schedule to collect retrospective data and we adopted DSM-5 criteria to define previous affective episodes. Lastly, another possible limitation is the exclusion of patients with comorbid substance abuse, which have reduced the generalizability of our findings. It has been reported that patient with severe mental disorder and comorbid substance use disorders presents more negative outcomes than their counterparts without comorbid disorders and presents more frequently a reduced psychological functioning [9]. However, we intended to recruit a sample with MD and comorbid substance abuse in order to compare impairments in psychological functioning among groups.

## Conclusions

Results of our study support the evidence that some psychological and temperamental characteristics are associated with functional impairment in mood disorders. Affective dispositions, quality of life, and trait-related impulsivity should be routinely assessed in ordinary practice, with the aim to identify patients with and increased risk to present psychological impairment and to develop personalized and targeted interventions. These interventions could be developed, also taking advantage from new technologies [72] and social media, which can booster the scalability of such interventions [32].

## Data Availability

Data presented in this study are available on request from the corresponding author on a reasonable request.
